# Position Paper: fragmented youth healthcare services in the Netherlands endanger treatment of teenage boys with psychiatric disorders

**DOI:** 10.1007/s00787-024-02378-x

**Published:** 2024-02-16

**Authors:** Rinske IJsselhof, Amy Hintjens, Anne Pelzer, Edward Nieuwenhuis

**Affiliations:** 1 Department of Primary and Long-Term Care, University Medical Center Groningen, Zwolle, The Netherlands; 2 University College Roosevelt, Middelburg, The Netherlands; 3 Department of Child and Adolescent Psychiatry, Reinier Van Arkel, Herlaarhof, Vught, The Netherlands; 4 Department of Pediatrics, University Medical Center Utrecht, Utrecht, The Netherlands

**Keywords:** Youth mental health care, Gender disparities, Psychiatric admissions, Cross-domain collaboration, Undertreatment in psychiatric care, Access to care disparities

## Abstract

For children who show strongly deviant behaviour in the Netherlands, a distinction is made between behavioural problems and psychiatric problems. As a result, two different domains have emerged over time, each with its own legal frameworks and inclusion and exclusion criteria. Consequently, there is no well-organized, coherent system for youth mental health care in the Netherlands. This strong dichotomy raises the question whether patients are being admitted to facilities where they are receiving appropriate care. In addition, referral bias can arise, because the type of complaint with which a young person presents is often dependent on the type of coping of the individual and thus, in turn, the gender of the patient. In this Position Paper, we examined the gender distribution at a youth psychiatric high and intensive care (HIC-Y) and other streams of youth care in the Netherlands to explore possible inequities in access to psychiatric care among children and adolescents. Results show that girls are significantly more likely than boys to be admitted to the HIC-Y for suicidal thoughts, self-harm and emotional dysregulation. In fact, girls account for 80% of all admissions, while boys account for only 20%. In contrast, regional and national reports from youth services and probation show a majority of boys being admitted (56–89%). The way care is organized (lack of cross-domain collaboration and the interplay between gender-dependent coping and exclusion criteria) seems to play a role in the underrepresentation of boys in acute psychiatry and their overrepresentation in secure youth care. Based on our research results, the concern is raised whether boys have a greater chance of undertreatment for psychiatric problems. Further research is needed to better understand the underlying factors that contribute to gender bias in psychiatric admissions, and to develop interventions that promote gender equality in healthcare.

## Introduction

The Netherlands lacks a coherent system for youth mental health care [1]. Specifically, there is a deep distinction between behavioural problems and psychiatric problems, leading to two different referral domains with their own legal frameworks and inclusion and exclusion criteria.

Children and adolescents with severe psychiatric symptoms have been treated in residential high and intensive care (HIC) units since 2017. To be admitted to those clinics they need to be referred by their general practitioner or another medical doctor (in case of voluntary care) or be sent by a judge (in case of involuntary care). The HIC model is unique to the Netherlands and was developed at the request of the government and the Dutch Association of Psychiatrists with the aim of improving the quality of inpatient mental health care and reducing coercion [2].

Children and adolescents with behavioural problems are educated in Youth Care services. Behavioural problems relate to behaviour that significantly harms the patient or his/her family/social network. Modification of the Youth Care Act in 2008 made it possible for judges to send teenagers with severe behavioural problems to closed residential facilities (secure youth care (involuntary care)) [3]. 61–85% of those patients are having psychiatric problems and meet criteria for DSM-5 diagnoses [4]. Van Dam and colleagues defined a psychiatric problem as a clinical overall score (*T* ≥ 64) at the Child Behaviour Check List (CBCL) at start of admission (T1).

Post-traumatic stress disorders and attachment disorders are among the most registered diagnoses (together with starting personality disorders or drug addictions). This percentage is substantial higher when compared to the general population [5] and is in line with recent findings in literature showing that internal problems can be under the surface of externalizing problems and that both types of problems can reinforce each other [6–8]. Once the teenager has been sent to Secure Youth Care it barely happens that he/she is being transferred to HIC youth (HIC-Y). Secure youth care cannot be seen as a substitute for psychiatric care, as it is not the focus. Teens are being educated and cared for. Research has shown that only 6% of teens with psychiatric symptoms who are admitted to secure youth care are transferred to psychiatric care after termination of secure youth care [9]. When both behavioural problems and psychiatric symptoms are present children may be placed into a very intensive and short-term observation and stabilisation ward (ZIKOS). This is only an option when the psychiatric symptoms are not the main long-term priority of care.

The profound distinction between behavioural care facilities and psychiatric care facilities raises the question whether patients are being admitted to the appropriate facilities. Especially, because referrals tend to focus on the main problem of the teenager, but this might be strongly influenced by coping strategies who are known to be dependent on gender. Girls tend to use more emotion-focused coping strategies which may conduct them to psychiatric care, while boys use more avoidant or solution-focused coping strategies (for example, vandalism, drug use and violence) which may conduct them to other services (probationary, youth care) [10–12].

In this Position Paper, a group of medical doctors working in child and adolescent psychiatry, paediatrics and primary care reflects on the impact of the current organisation of care on the accessibility of psychiatric care for teenage boys and girls. We assessed the gender distribution at an HIC-Y, as well as other streams of youth services in the Netherlands, to explore possible inequalities of access to psychiatric care that children and adolescents may be experiencing.

### Patient population

We performed a retrospective single centre review of patients who were admitted to the HIC-Y Herlaarhof Vught between January 1, 2020, and December 31, 2022 and obtained demographic data. Patients were aged 12–18 years with severe psychiatric symptoms in need of admission to our acute psychiatric ward. Both patients who lived inside and outside our region were included. Admission data of the HIC Y was retrieved from the electronic patient record and was anonymized for processing by one of the authors. The number of all admissions was used for our analysis and this was not corrected for the number of unique patients. In other words, when a patient was admitted several times, all admissions were added to the database. When correcting for the number of unique patients the same distribution was seen, since readmissions occurred rarely and were seen in both boys and girls. Patients with gender dysphoria were not excluded, but sex assigned at birth was used for the analysis.

### Outcomes of interest

The outcome variables that were analysed were the following:

• Primary: sex assigned at birth.
• Secondary: age, city of residence (within or outside our region).

### Statistical analysis

Patient and procedural characteristics were summarized as frequencies and percentages for categorical variables and medians and interquartile ranges (IQR) for continuous variables. The proportion of our outcome, sex assigned at birth, was calculated for our cohort. A confidence interval (CI) was used to determine whether the proportion of our cohort significantly differed from the proportion of the Dutch population where 51% of children and adolescents between 12 and 18 years were boys [13].

The following formulas were used for calculating the proportion, standard error and 95% CI of the proportion:

• proportion (*p*) = boys / *n*.
• standard error (Se)(*p*) = √((p*(1-*p*)/*n*)).
• 95% CI (*p*) = *p* – 1,96* (se)(*p*) to *p* + 1,96*Se(*p*).

## Results

433 patients were included in the analysis. There were 86 boys (20%). The median age was 16 years. There were 235 patients (54%) from outside our region. Patient characteristics are shown in Table 1.

**Table 1:** Patient characteristics (*n* = 433)

Characteristic	Value [number (percentage) or median]
Male	86 (20%)
Age, years	16
Admissions from outside region	235 (54%)

The male:female ratio was assessed using the following equations:

•*p* = 86 / 433 = 0,20.
•se (0,20) = √((0,20*(1–0,20)/433) = 0,019.
•95% CI (0,20) = 0,20–1,96 * 0,019 to 0,20 + 1,96 * 0,019.
•95% CI (0,20) = 0,16 to 0,24.

The 95% CI of our proportion (0,16 to 0,24) shows a strong significant difference when compared to the proportion of the population (0,51). There are significantly less boys admitted to our HIC-Y when compared to the percentage of teenage boys in the Dutch population [13].

### Other referral patterns of youth services

Distribution of sex at birth for admissions to youth care and probationary services are shown in Table 2. The majority of patients admitted to secure youth care were boys on both a regional level (60% in the north–west and 56% in the south–west) and a national level (56%). The gender distribution at a ZIKOS ward, where youth receive intensive psychiatric care, the majority of admissions were girls for both the north–west (70%) and south–west regions (72%). In contrast, the majority of admissions to probationary services was male (84% overall), especially in the voluntary probationary services (89%).

**Table 2:** Comparison with other streams of youth services

Admissions 2020–2022	Boys (percentage)
HIC-Y Vught (*n* = 433)	86 (20%)
Secure youth care in the north–west (*n* = 476)	285 (60%)
ZIKOS (*n* = 10)	3 (30%)
Secure youth care in the south–west (*n* = 495)	277 (56%)
ZIKOS (*n* = 94)	26 (28%)
National secure youth care (*n* = 2080)	1160 (56%)
National probationary services (*n* = 8555)	7150 (84%)
Involuntary (*n* = 7885)	6565 (83%)
Voluntary (*n* = 2025)	1800 (89%)

## Discussion

The results show that girls were significantly more likely than boys to be admitted to the HIC-Y Herlaarhof for suicidal ideation, self-harm and emotional dysregulation. In fact, girls accounted for 80% of all admissions, while boys accounted for only 20%. The majority of these admissions (54%) were placements of patients from both central and southern parts of the Netherlands (large areas outside our region) so the results seem to reflect the national situation. Interestingly, regional and national reports of youth care services and probationary services show a majority of boys being admitted (56–89%).

Based on findings in literature we think that personal (diagnosis and coping strategies) and organisational factors may play a role in the underrepresentation of boys in the HIC-Y ward and their overrepresentation in secure youth care and probationary services [10–12, 14–17].

### Personal factors

The question is raised how the experienced entrapment of the HIC-Y population relate to specific diagnoses. Literature shows that symptoms in people with psychiatric disorders may differ by gender [14–16]. Women with autism–spectrum disorder or attention–deficit hyperactivity disorder are believed to be underdiagnosed, and have been long neglected in psychiatric care compared to male peers, even when meeting the criteria for diagnosis [16]. This hints at a gender bias in psychiatry, meaning it is vital to remain vigilant for any subpopulations being underrepresented in care [14].

Since the HIC-Y accepts all patients with emotional dysregulation and suicidal behaviour, regardless of DSM diagnoses, the observed gender inequity cannot entirely be explained by a different incidence of psychiatric disorders, although timing can play a role. For instance, if girls with autism are underdiagnosed during infancy, they may be overrepresented during teenager years.

Moreover, our literature search of coping strategies revealed that girls tended to use more emotion-focused coping strategies which may conduct them to psychiatric care, while boys used more avoidant or solution-focused coping strategies (for example, vandalism, drug use and violence) which may conduct them to youth care and probationary services and keep them excluded from psychiatric care since these are exclusion criteria for many institutions [10–12].

### Organisational factors

**Fig. 1:**
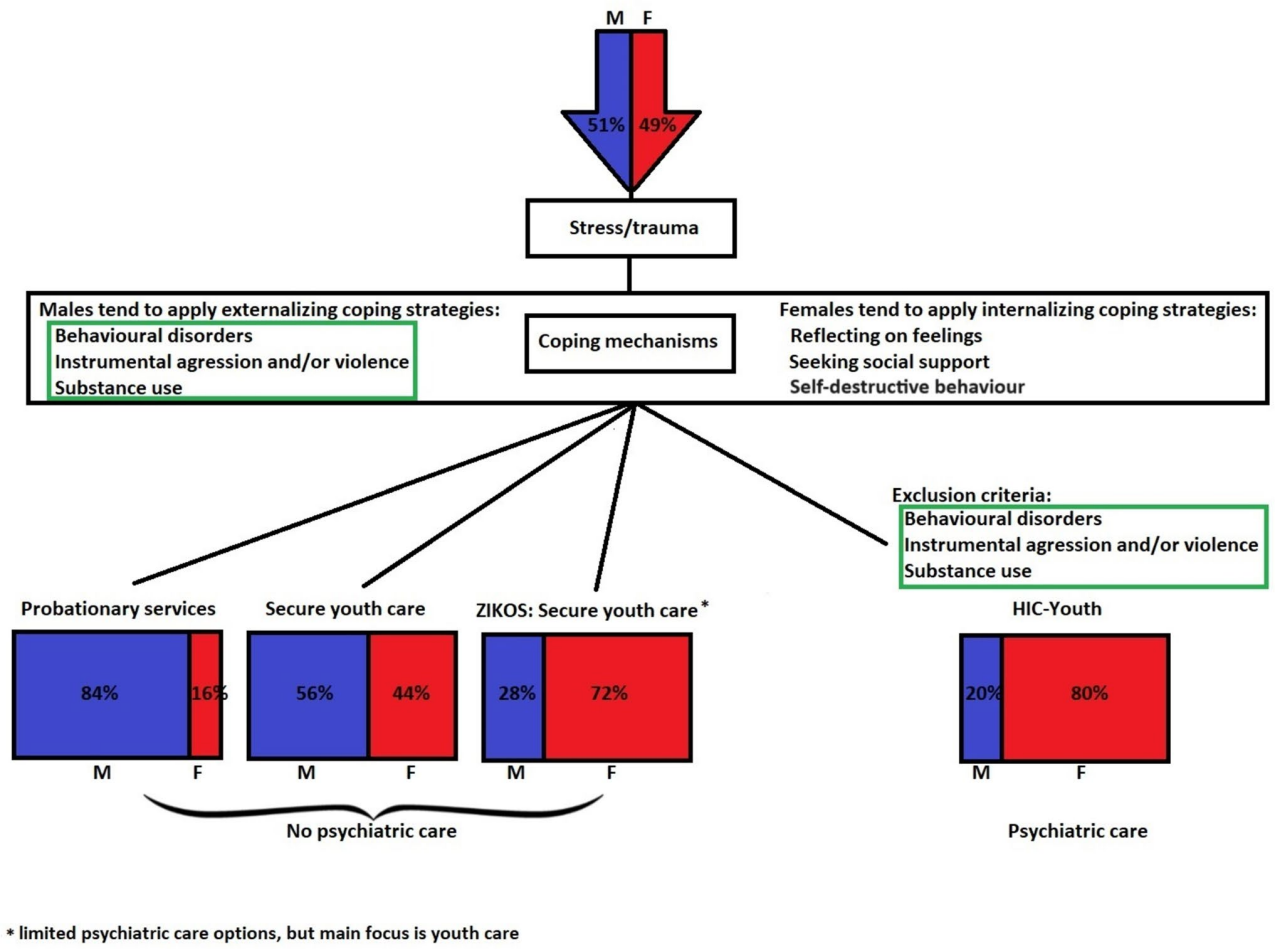
Exclusion criteria and gender disparities in access to psychiatric care: a comparative analysis, highlighting gendered coping strategies

HIC-Y are known to have exclusion criteria (see Appendix I). When behaviour disorders are the main focus or in case of instrumental aggression/violence or substance use, the teenagers is excluded from admission to the HIC-Y where psychiatric care is the main focus. Since the above-mentioned factors are coping mechanisms associated with males the question has been risen whether the way care is organized has driven boys with underlying psychiatric disorders into youth care and probationary services instead of psychiatric care (Fig. 1).

**Fig. 2:**
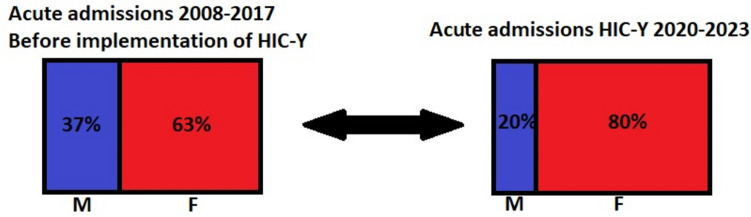
Gender distribution in youth psychiatric clinics pre- and post-implementation of HIC-Y model in the Netherlands

A skewed gender distribution at acute youth psychiatric clinics (boys 37%) was already seen in the Netherlands before the implementation of the HIC-Y model in 2017 [17]. After implementation of the HIC-Y, we see a 30% further decline of male admissions (boys 20%) (Fig. 2).

Our observation of underrepresentation of boys (20%) at the HIC-Y differs from data of adult HIC units where only a slight majority of patients being admitted is male (56.1%) [18]. This emphasizes the institution of youth care as part of the care may underlie this. Once boys pass the age of 18, they are no longer eligible for juvenile care and are more readily admitted to adult HIC units.

In other countries, where youth mental health care is being organised differently, gender differences in admission rates are less extreme. A systematic review and meta-analysis found no association between sex and involuntary hospitalization in children and adolescents [19]. Two recent large studies from Jendreyschak et al. and Kandsperger et al., colleagues from our neighbouring country Germany, show no big gender difference in admission rates for teenagers, with boys accounting for, respectively, 57% and 41% of admissions to acute psychiatry wards [20, 21].

A recent Dutch study among General Practitioners dealing with psychosocial problems of children and youth in the Netherlands shows that they refrain from considering and consulting interdisciplinary teams if they had negative collaboration experiences, even if this choice resulted in poorer quality of care [22].

This finding corresponds with another study about cross domain collaboration in which poor communication, trust and support resulted in perceived patient delay [23]. These findings underline the importance of interprofessional collaboration as a key factor in initiatives designed to increase the effectiveness of health services [24].

## Conclusion

The way youth health care is organized in the Netherlands seems to cause gender inequality. The current care system is divided between secure youth care and psychiatric care with a lack of cross-domain collaboration. Psychiatric care units apply exclusion that refer to coping strategies associated with the patient’s gender and can be mistaken for ‘mere’ behavioural problems, yet are only the surface of underlying severe psychiatric illness like depression. Both the lack of cross-domain collaboration and the relationship between gender-dependent coping and exclusion criteria appear to play a role in the underrepresentation of boys in acute psychiatry and their overrepresentation in secure youth care. We hope this paper will raise awareness among (referring) healthcare professionals and policy makers.

Further research is needed to better understand the underlying factors contributing to gender bias in psychiatric referrals and to develop interventions that promote gender equity in health care.

## Data Availability

Data availability statement excluded due to confidentiality requirements.

## Appendix I: An overview of the inclusion and exclusion criteria of patients admitted to the HIC-Y unit.

**Table:**

Inclusion criteria	Exclusion criteria
The patient is between the ages of 12 and 18 years In cases of voluntary admission, there should be signs of a serious (and systematic) psychiatric crisis. Intensive out-patient care would be insufficient to halt regression or break the negative spiral In cases of involuntary admission, the patient must be admitted according to the law of the *Geestelijke Gezondheidszorg (GGZ)*, which is the public mental health care system in the Netherlands. Patients may be admitted via a crisis order, or a care order Patients must have a residence to return to once they are discharged Before admission, there must be an agreement made on goals that are realistic within the timeframe of care. This is done for the sake of preparation and management of expectations A chain of caregivers such as guardians and municipalities are involved from admission to ensure continuity and embeddedness of care	The patient has active substance use The patient has comorbid somatic symptoms that require medical intervention The patient has a cognitive impairment that requires a different treatment environment and treatment plan than the HIC can provide. In such cases, a referral to an *LVB* setting (a Dutch service that provides care for people with a slight mental handicap) is advised The patient has a personality disorder with a high chance of regression and hospitalisation. These patients may only be admitted under very strict criteria on a short-term basis, given that the HIC care is provided as part of a long-term outpatient treatment plan A pedagogical or systematic crisis is the main focus (a psychiatric crisis is not the main reason for admission). In this case, intensive out-patient treatment with a chain of caregivers or foster care is indicated The patient shows signs of instrumental aggression and/or violence
